# Monocytes as Targets for Immunomodulation by Regional Citrate Anticoagulation

**DOI:** 10.3390/ijms25052900

**Published:** 2024-03-01

**Authors:** Giovana Seno Di Marco, Achmet Imam Chasan, Göran Ramin Boeckel, Katrin Beul, Hermann Pavenstädt, Johannes Roth, Marcus Brand

**Affiliations:** 1 Department of Internal Medicine D, University Hospital Muenster, 48149 Muenster, Germanymarcus.brand@ukmuenster.de (M.B.); 2 Institute of Immunology, University of Muenster, 48149 Muenster, Germany

**Keywords:** anticoagulation, citrate, heparin, monocytes, hemodialysis, immunomodulation

## Abstract

Immune alterations in end-stage renal patients receiving hemodialysis are complex and predispose patients to infections. Anticoagulation may also play an immunomodulatory role in addition to the accumulation of uremic toxins and the effects of the dialysis procedure. Accordingly, it has been recently shown that the infection rate increases in patients under regional citrate anticoagulation (RCA) compared with systemic heparin anticoagulation (SHA). We hypothesized that RCA affects the immune status of hemodialysis patients by targeting monocytes. In a cohort of 38 end-stage renal patients undergoing hemodialysis, we demonstrated that whole blood monocytes of patients receiving RCA—but not SHA—failed to upregulate surface activation markers, like human leukocyte antigen class II (HLA-DR), after stressful insults, indicating a state of deactivation during and immediately after dialysis. Additionally, RNA sequencing (RNA-seq) data and gene set enrichment analysis of pre-dialysis monocytes evidenced a great and complex difference between the groups given that, in the RCA group, monocytes displayed a dramatic transcriptional change with increased expression of genes related to the cell cycle regulation, cellular metabolism, and cytokine signaling, compatible with the reprogramming of the immune response. Transcriptomic changes in pre-dialysis monocytes signalize the lasting nature of the RCA-related effects, suggesting that monocytes are affected even beyond the dialysis session. Furthermore, these findings demonstrate that RCA—but not SHA—impairs the response of monocytes to activation stimuli and alters the immune status of these patients with potential clinical implications.

## 1. Introduction

End-stage renal disease (ESRD) is associated with significantly increased morbidity and mortality. The leading causes of death in these patients are cardiovascular and infectious diseases, which are pathological processes related to immune dysfunction. On the one hand, a chronic low-grade inflammation state and the activation of immune cells, which generally characterize patients with ESRD, increase the risk of atherosclerosis. On the other hand, a poor host response increases susceptibility to infections [1,2].

Immune alterations in ESRD are complex and multifaceted. Among the cells involved, monocytes appeared to be mainly dysregulated, showing a high level of basal inflammation combined with impaired activation upon infectious challenge [3,4]. The accumulation of uremic toxins and the effects of renal replacement therapy per se may be involved in these alterations [5]. Uremic toxins are associated with both the activation (surface marker profile and cytokines) and functional impairment of monocytes, while the dialysis procedure is mainly related to their activation [3,6,7]. Contributing to this effect are several factors, like removal efficiency of uremic solutes, interactions between blood and dialysis membrane, endotoxin contamination in solutions, and access-related infections [7,8,9].

Additionally, the anticoagulation modality may also play an immunomodulatory role. Even though heparin is the anticoagulant of choice in intermittent hemodialysis (iHD) procedures, regional citrate anticoagulation (RCA), generally used in continuous renal replacement therapy for critically ill patients, has appeared to be an effective and safe alternative for hemodialysis patients at high risk of bleeding [10,11,12,13]. However, Zarbock et al. have recently shown more adverse events regarding metabolic complications and an increased rate of new infections in patients with acute kidney injury receiving RCA compared with systemic heparin anticoagulation (SHA) [14].

Here, we showed that RCA induces immunomodulatory effects on monocytes from ESRD patients receiving iHD compared to SHA. By combining information on surface marker expression, transcriptomics, and cytokine production, we describe the partial unresponsiveness and deactivation of monocytes to stimuli during and beyond the dialysis session.

## 2. Results

### 2.1. Cohort Characteristics

**Figure 1:**
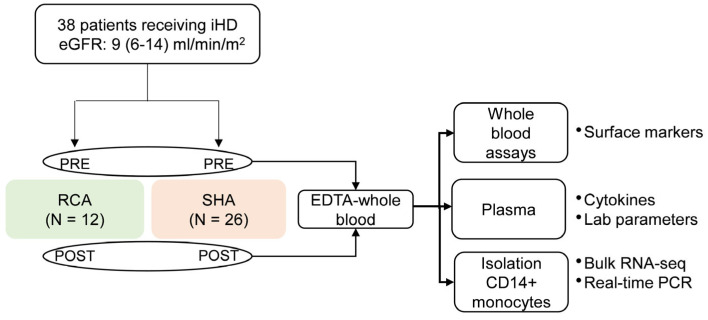
Overview of study workflow. Schematic representation of study design. Citrate, ESRD patients receiving regional citrate anticoagulation (RCA); heparin, ESRD patients receiving systemic heparin anticoagulation (unfractionated heparin); ESRD, end-stage renal disease; iHD, intermittent hemodialysis; RNA-seq, RNA sequencing; PCR, polymerase chain reaction; pre, pre-dialysis, before anticoagulation; post, immediately after dialysis.

The study cohort consisted of thirty-eight ESRD patients receiving iHD randomized into two groups: twelve receiving RCA and twenty-six receiving SHA. Data obtained from twenty-five healthy subjects were used as a reference (control) for some experiments. A flowchart for patient enrollment and study design is depicted in Figure 1. On average, patients in the citrate group received six dialysis sessions (4 [3, 14]; median [min, max]) by the day of sample collection. Patients in the SHA group received heparin for more than three months. Baseline demographics and clinical and laboratory characteristics of the study participants are given in Table 1. There are no differences regarding the degree of uremia (serum creatinine, blood urea nitrogen) or dialysis efficacy (Kt/V) between the two groups of patients, yet pre-dialysis plasma phosphate levels are significantly lower in the RCA group.

### 2.2. Differential Expression of Activation Markers on iHD Patient Monocytes Respective to the Anticoagulation Modality

**Figure 2:**
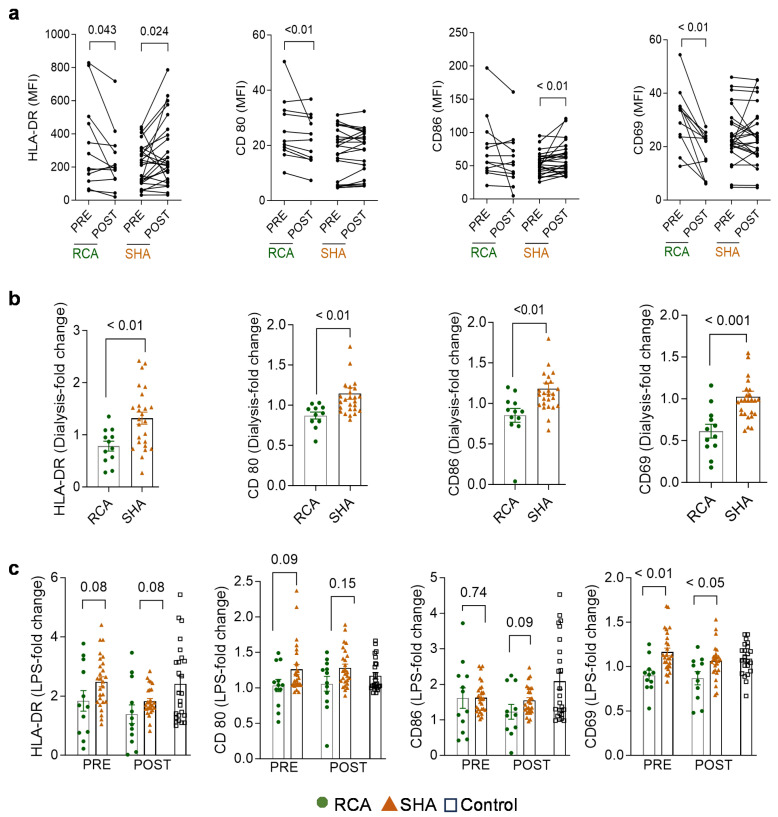
Effect of the dialysis procedure and lipopolysaccharide (LPS) stimulation on the expression of surface activation markers on blood monocytes from ESRD patients receiving regional citrate anticoagulation (RCA) or systemic heparin anticoagulation (SHA). (**a**) Whole blood was collected from patients before (pre) and after (post) intermittent hemodialysis. The graphs show the mean fluorescence intensity (MFI) of the specific markers measured by multicolor flow cytometry. (**b**) Changes in monocyte surface marker expression in post-dialysis blood relative to pre-dialysis. (**c**) Whole blood was incubated in the presence or not of 2 ng/mL LPS for 4 h before flow cytometry analysis. The graphs show the changes in the expression in LPS-treated relative to non-treated monocytes. Data obtained from healthy subjects were used as reference (control) only. A Wilcoxon matched-pairs signed rank test ((**a**), pre vs. post) or a Mann–Whitney U test ((**b**,**c**), RCA vs. SHA) was applied. ESRD, end-stage renal disease; HLA-DR, human leucocyte antigen–DR.

As a measure of host immune function and monocyte activation, we compared the expression of early (CD69) and late (HLA-DR) activation markers and costimulatory molecules (CD80, CD86) on CD14^+^- monocytes before and after a hemodialysis session. Multicolor flow cytometric analysis of the whole blood showed no significant differences in the expression level (measured as mean fluorescence intensity, MFI) of these surface markers between the two groups of patients before dialysis. Interestingly, most markers were upregulated after an iHD session using SHA, while they were downregulated after RCA-based iHD (Figure 2a). Figure 2b shows the fold-change expression of the surface markers after dialysis (relative to pre-dialysis) and evidences the differences between the two anticoagulation modalities regarding the ability of monocytes to regulate the expression of their activation markers.

Furthermore, monocytes from patients receiving RCA failed to increase the expression levels of activation markers, particularly HLA-DR and CD69, in response to LPS exposure (2 ng/mL for 4 h) to the same extent as monocytes from patients receiving SHA. Interestingly, these differences were observed in monocytes obtained from blood samples collected before (pre) and after (post) an iHD session (Figure 2c), suggesting that RCA’s effects may extend beyond the dialysis session.

### 2.3. Transcriptional Changes in Monocytes of Patients Receiving RCA and SHA

**Figure 3:**
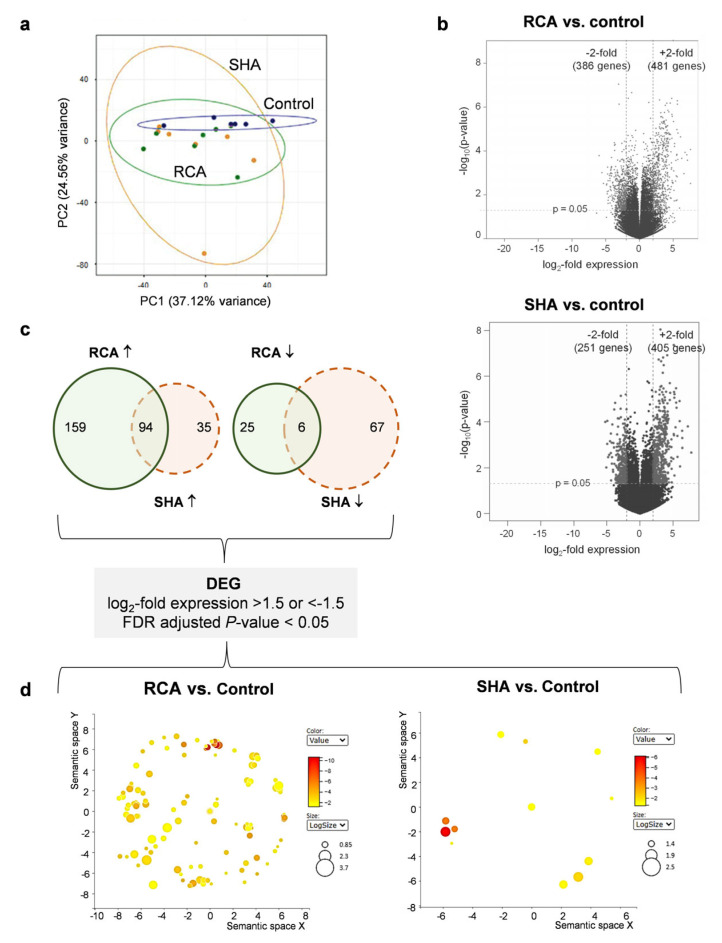
Transcriptomic profiling of pre-dialysis monocytes isolated from ESRD patients from ESRD patients receiving regional citrate anticoagulation (RCA) or systemic heparin anticoagulation (SHA). Blood samples were collected before dialysis, and isolated monocytes were left to rest overnight before RNA isolation. (**a**) Principal component (PC) analysis of bulk RNA-seq data of cells isolated from patients receiving RCA (n = 7) or SHA (n = 7) and healthy control subjects (n = 6). Each dot represents one subject, and the variance percentage is shown in brackets. (**b**) Volcano plot representation of differential expression analysis calculated by DESeq2 package. Each dot represents a single gene. Significantly regulated genes (log2-fold expression > 2 or <−2, and *p*-value < 0.05) are highlighted in gray. (**c**) Venn diagram showing the number of shared differentially regulated genes in patient monocytes relative to control monocytes (left, upregulated genes; right, downregulated genes). Only significant genes with a log2-fold expression > 1.5 or <−1.5 and false discovery rate (FDR)-adjusted *p*-value < 0.05 were included. (**d**) REVIGO scatterplots showing clusters representative of enriched gene ontology biological process terms. The color indicates the log_10_(*p*-value), and the size indicates the number of annotations for the term in the gene ontology annotation database. ESRD, end-stage renal disease.

To identify long-term transcriptional changes in patient monocytes and the effects of the different anticoagulation modalities on these changes, we performed bulk RNA-seq in monocytes isolated from pre-dialysis blood samples obtained from iHD patients and control participants, used as a reference group. Further, we compared the RCA group vs. control and the SHA group vs. control. Principal component analysis revealed moderate separation between the two patient cohorts (Figure 3a). Compared to controls, we found 481 vs. 405 upregulated and 386 vs. 251 downregulated transcripts in monocytes isolated from patients receiving RCA and SHA, respectively (log2-fold expression > 2 or <−2, and *p*-value < 0.05) (Figure 3b). By applying a more conservative, adjusted analysis, we found a more pronounced difference between RCA and SHA groups: 253 vs. 129 upregulated and 31 vs. 73 downregulated transcripts, respectively (log2-fold expression > 1.5 or <−1.5, and false discovery rate (FDR) adjusted *p*-value < 0.05) (Figure 3c). RNA-seq data are given as primary data sets 1 and 2 (excel spreadsheet). The DEGs subsequently analyzed by gene set enrichment approaches were chosen according to the FDR adjusted *p*-value < 0.05 and log2-fold expression > 1.5 and <−1.5.

**Figure 4:**
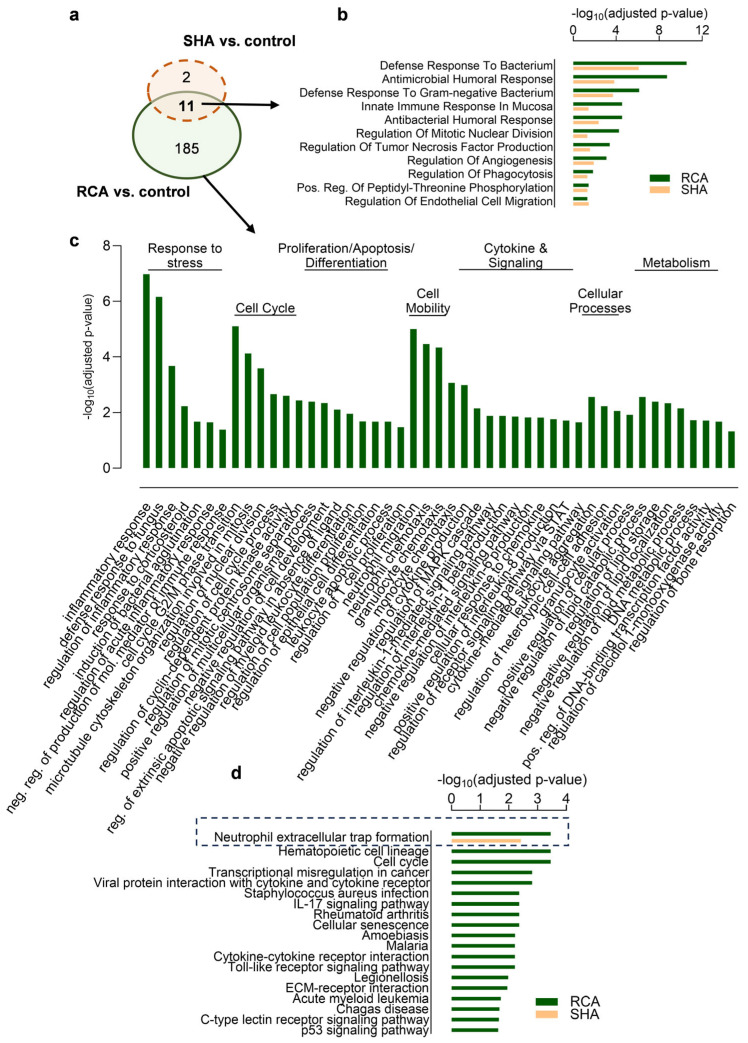
Top enriched gene ontology hits for biological process 2023 and KEGG 2021 human pathways for the differentially expressed genes (log2-fold expression > 1.5 and adjusted *p*-value < 0.05) in patient monocytes relative to control. (**a**) Venn diagram showing the number of enriched GOBP terms in patient monocytes relative to control. Bar charts showing (**b**) the GOBP terms shared between RCA and SHA groups, (**c**) representatives of terms exclusively enriched in the RCA group (see also Table S1), and (**d**) enriched KEGG pathways. The terms are displayed based on the −log_10_(adjusted *p*-value) according to the data obtained from ENRICHR analysis. The dashed box indicates the single enriched KEGG pathway shared between RCA and SHA groups. RCA, regional citrate anticoagulation; SHA, systemic heparin anticoagulation.

Gene ontology biological process (GOBP) analysis evidenced the great difference between the RCA (196 biological processes, adjusted *p*-value < 0.05, Table S1) and SHA (13 biological processes, adjusted *p*-value < 0.05, Table S2) groups as summarized by the REVIGO scatterplot (Figure 3d and Figure 4a). Upregulated genes in monocytes obtained from patients undergoing iHD (relative to controls) were substantially associated with biological processes and pathways related to the immune system and defense and antimicrobial responses irrespective of the anticoagulation modality, given that the related GOBP terms are overrepresented in the RCA with high significance levels (Figure 4b). Additional terms related to the inflammatory response/response to stress (e.g., “regulation of inflammatory response” and “negative regulation of production of molecular mediator of immune response”), cell cycle, proliferation, and differentiation (e.g., “negative regulation of myeloid leukocyte differentiation” and “leukocyte apoptotic process”), cytokine and signaling (e.g., “negative regulation of cytokine production”), cell mobility, cellular processes, and cell metabolism (e.g., “regulation of calcidiol1-monooxygenase activity”) are solely enriched in the RCA group (Figure 4c). The complete list of significantly enriched GOBP terms and related genes is given in Table S1. KEGG (Kyoto Encyclopedia of Genes and Genomes) analysis indicated that, in addition to the “neutrophil extracellular trap formation”, also enriched in the SHA group, the top pathways in the RCA group are related to cell cycle, hematopoietic cell lineage, cytokine signaling, transcriptional regulation, and disease pathways (Figure 4d). Reactome analysis underlined the enrichment of pathways related to the regulation of the cell cycle and p53 signaling pathway (Figure S1), given that the analysis of upstream regulators by Ingenuity Pathway Analysis predicted inhibition of the p53 pathway with an activation z-score of −2.325 and an overlap *p*-value of 2.56 × 10^−21^. Pathway and ontology analysis found no significant (adjusted *p*-value < 0.05) terms associated with the set of transcripts downregulated in both patient groups compared to controls.

**Figure 5:**
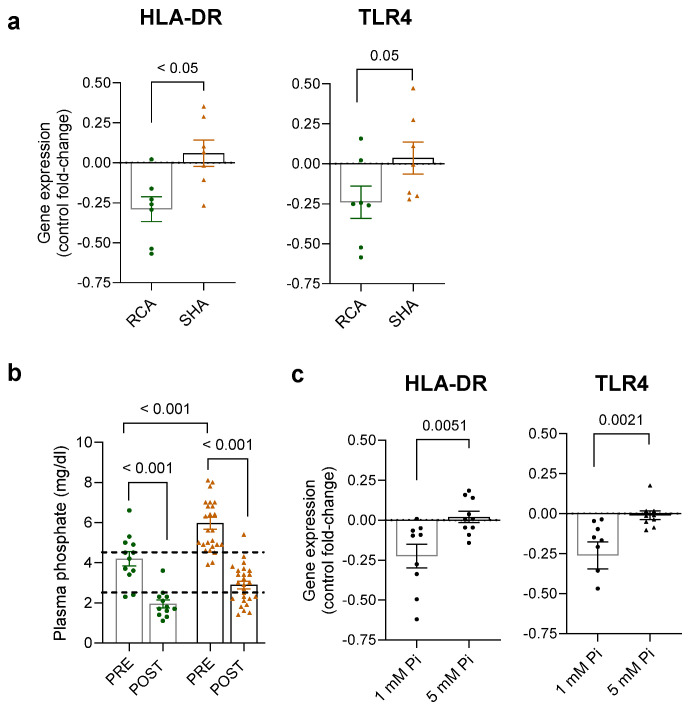
Gene expression analysis and plasma changes in ESRD patients receiving regional citrate anticoagulation (RCA) and systemic heparin anticoagulation (SHA). (**a**) Blood samples were collected before dialysis, and isolated monocytes were left to rest overnight before RNA isolation. Graphs show real-time PCR data of cells isolated from patients receiving RCA or SHA relative to healthy control subjects (n = 6). (**b**) Phosphate levels in plasma samples before (pre) and after (post) intermittent hemodialysis. Dashed lines indicate the lower and upper limits of the phosphate reference range. One-way analysis of variance was applied. (**c**) THP-1 monocytes were cultured in a medium containing 1 and 5 mM phosphate for three days, and gene expression was analyzed by real-time PCR. Graphs show expression fold change relative to high Pi. In (**a**,**b**), results are log-transformed and given as mean ± SEM. The Mann–Whitney U test was applied. ESRD, end-stage renal disease; HLA-DR, human leucocyte antigen–DR; MCP-1, monocyte chemoattractant protein-1; PCR, polymerase chain reaction; Pi, phosphate; TLR4, toll-like receptor 4.

Interestingly, complementary gene expression analysis by real-time PCR evidenced a decreased expression of HLA-DR in the monocytes isolated from the RCA compared to the SHA group (Figure 5a). Toll-like receptor 4 (TLR4), a critical pattern recognition receptor, was also decreased in the same group on the borderline of statistical significance. The groups did not differ regarding the expression levels of tumor necrosis factor-α and interleukins 1β, 6, and 8 in the isolated monocytes.

### 2.4. Systemic Changes Associated with the Anticoagulation Modalities

To characterize the differences in the environment that bathes circulating monocytes, we determined the profile of critical proinflammatory cytokines and other factors in the pre-dialysis patient’s plasma by immunoassays (Table 2 and Figure 5). The level of inflammation estimated by C-reactive protein (CRP; Table 1) and interleukin-6 (IL-6; Table 2) are similar. Plasma phosphate was lower in the RCA compared to the heparin group at the time of pre-dialysis, and the levels further declined at post-dialysis (Figure 5b). Strikingly, THP-1 cells (human monocytic cells) cultured in a low-phosphate medium for three days displayed decreased gene expression of HLA-DR and TLR4, as did monocytes isolated from the RCA group (Figure 5c), suggesting that lower phosphate in RCA (compared to SHA) could have played a role in the lower HLA-DR and TLR4 expression.

Among the antimicrobial peptides analyzed, β-defensin-3 is diminished in the RCA group (*p* = 0.0583 vs. heparin) (Table 2).

Furthermore, we also observed decreased levels of fibroblast growth factor 23 (FGF23) levels in the RCA group (*p* < 0.001 vs. heparin), remembering that FGF23 is a potent phosphate regulator associated with metabolic changes, endothelial dysfunction, and cardiovascular disease in renal patients (Table 2).

## 3. Discussion

In the current study, we combined information on surface marker expression and transcriptomics in CD14^+^- monocytes and changes in plasma cytokines and other factors to unveil distinct immunomodulatory effects of RCA and SHA in patients receiving iHD. As a measure of immune status, we compared the expression of early (CD69) and late (HLA-DR) activation markers and costimulatory molecules (CD80, CD86) on blood CD14^+^- monocytes before and after an iHD session. Although the baseline expression (pre-dialysis levels) of surface markers did not differ between the citrate and heparin groups, our data evidenced the impaired response of monocytes from patients receiving RCA to activation stimuli, like the dialysis procedure or LPS stimulation.

Among cell surface changes, decreased HLA-DR expression on monocytes is a hallmark of functional deactivation of monocytes and immune incompetence in patients after stressful insults (e.g., trauma, severe surgery, sepsis), predisposing them to late infections and poor clinical outcomes [15,16]. Half of our patients receiving RCA displayed a > 20% decrease in HLA-DR post-dialysis. We did not determine when HLA-DR returns to the baseline (pre-dialysis) levels after dialysis termination. Although a rapid return could mean a favorable prognosis [16,17], it is noteworthy that patients receiving iHD thrice weekly experience such changes regularly for several weeks. Furthermore, not only was HLA-DR expression altered, but also the costimulatory molecules CD80 and CD86. Conversely, impaired expression of these molecules on monocytes has already been described as a crucial aspect of the immune defect in chronic hemodialysis patients [18].

Dialysis is a complex procedure that regulates the levels of different molecules capable of direct or indirect modulation of HLA-DR expression [9]. In the current study, all patients were subjected to the same dialysis procedure (iHD) using the same type of dialyzer and equivalent dialysis efficacy, but the anticoagulation modality differed. So, it is highly likely that RCA use may have influenced the changes in the phenotypic profile of monocytes described above. Conversely, a decreased expression of the activation marker CD11b was observed in neutrophils from acute kidney injury patients receiving RCA-based continuous renal replacement therapy compared to patients receiving SHA [19]. Additionally, Ashbrook et al. have shown that citrate modulates the inflammatory responses in monocytes due to intracellular calcium chelation and changes in the cellular metabolism, e.g., alterations in gene transactivation [20].

In this scenario, we could attribute the lack of surface marker upregulation in post-dialysis monocytes from patients receiving RCA to the calcium-chelating effects of citrate. However, we should remark that even pre-dialysis monocytes responded to the LPS stimulation to a lesser extent than monocytes from patients receiving SHA. On average, patients in the citrate group were exposed to six RCA-based dialysis sessions by the day of sample collection. Therefore, we speculated that citrate-based anticoagulation over several days/weeks could lead to long-term alterations in monocyte gene expression and systemic cytokine profile.

Gene set enrichment analysis evidenced a great and complex difference between RCA and SHA groups regarding transcriptional changes. Minimal transcriptional differences were detected in monocytes isolated from the SHA group relative to controls. Both RCA and SHA groups present with an increased number of transcripts involved in the immune system and antimicrobial/bacterial responses, which may reflect the high rate of infections and changes in immune cell function inherent to the uremic conditions [21]. Additionally, monocytes displayed a dramatic transcriptional change in the RCA group, with elevated genes involved in the cytokine signaling, cellular metabolism, and cell cycle/proliferation, compatible with the reprogramming of the immune response and a state of immune tolerance that can increase the susceptibility to infection and the risk of adverse outcomes [22,23,24]. The enrichment of pathways associated with cell cycle/apoptosis and hematopoietic cell lineage may also indicate changes in hematopoietic cells’ self-renewal and differentiation capacities in the RCA group. In combination with the results discussed below, processes like “neutrophil migration”/”monocyte chemotaxis”, “negative regulation of cytokine production”, and others directly or indirectly associated with energy and intermediate metabolites conceivably show the dysfunction of monocytes connected with metabolic reprogramming or at least a high demand for compensatory mechanisms that were not found in the SHA group. The negative regulation of the p53 signaling pathway found in the RCA group may point to an underlying molecular mechanism. In addition to regulating cell proliferation and apoptosis, p53 controls the expression of many genes involved in cellular metabolism and immunity, playing a protective role in infectious diseases [25,26,27]. Therefore, its inhibition may indicate a defective immune system in the RCA group. In addition, the reduced expression of TLR4 and HLA-DR, the first line of defense for pathogen recognition in infectious diseases, corroborates the idea of a state of immune incompetence created under citrate anticoagulation.

Changes in plasma cytokines and other mediators, i.e., changes in the environment that bathes circulating monocytes, could explain the differences between the two patient groups observed herein. Remarkably, plasma phosphate levels were lower in the RCA group at the time of pre-dialysis. Even though these patients had no overt hypophosphatemia, they presented with very low phosphate levels post-dialysis. Low phosphate concentrations can limit energy metabolism in immune cells, cause leukocyte dysfunction, and increase the risk of infection [28,29]. Notably, citrate anticoagulation has already been shown to be associated with low phosphate levels/hypophosphatemia in other clinical studies [14]. MCP-1 is also decreased in the RCA group compared to the heparin group. In addition to chemotactic properties, this cytokine has significant pleiotropic effects on the immune system, ranging from monocyte activation and priming to infection to cell differentiation [30]. The depletion of plasma levels of MCP-1 during heparin-free hemodialysis has already been described elsewhere, even though an HD session with systemic heparinization usually increases MCP-1 levels [31,32].

Despite the changes implying that RCA may favor infection, the decreased levels of inflammatory markers (particularly MCP-1) and risk factors (e.g., plasma phosphate and FGF 23) could also indicate that patients receiving RCA are at lower risk of cardiovascular disease than patients receiving SHA [31,33,34,35,36]. In addition, it is noteworthy that our patients have received unfractionated heparin. Even though its antimicrobial, anti-inflammatory, and mucoactive activities are shared by low molecular weight heparin (LMWH), unfractionated heparin appears more potent than low molecular heparin regarding, for example, anti-viral effects [37,38]. Further research is needed to explore these topics (lower cardiovascular risk under RCA and differences between unfractionated and LMW heparin) more extensively.

We know that the small number of patients is an important limitation of our study, as well as the lack of validation of RNA-seq data through real-time PCR. However, our findings address an underexplored aspect of anticoagulation that can affect many patients receiving renal replacement therapy and add to other evidence showing RCA’s immunomodulatory effects on different immune cells [19,20,31,39]. The mechanisms behind these effects are still unclear. Yet, based on the nature of the systemic changes reported and the transcriptomic changes observed, it is reasonable that the regular use of RCA leads to the disruption of the normal cellular metabolism and physiological processes rather than direct immunosuppression itself.

Dialysis treatment puts patients at higher risk for infections and death. Irrespective of the anticoagulation of choice, immune monitoring (e.g., quantification of HLA-DR expression) and a better follow-up may be valuable attempts to improve outcomes and individualize target therapy strategies (e.g., antibiotics and immunomodulators) in these patients [40,41,42,43].

## 4. Materials and Methods

### 4.1. Study Population

The protocol was approved by the Joint Ethics Committee of the *Landesärztekammer Westfalen-Lippe* and the Medical Faculty of the University of Münster, Münster, Germany (No. 2018-300-f-S) and adhered to the Declaration of Helsinki. In addition, written informed consent was obtained from all patients. We performed a longitudinal study with 38 ESRD patients enrolled from August 2018 to September 2019 at the Department of Internal Medicine D, University Hospital Muenster. Consecutively attending patients (age > 18 years) undergoing iHD treatment were included. Twenty-six patients received SHA; twelve patients at high risk of bleeding or with a history of hypersensitivity reactions against heparin received RCA. Data obtained from twenty-five healthy subjects were used as a reference (control) for some experiments. Exclusion criteria were current infection and malignancy, a history of organ transplantation and immunosuppression, and pregnancy. All patients were maintained on their regulation medication.

### 4.2. Intermittent Hemodialysis and Anticoagulation Procedure

Intermittent hemodialysis sessions were performed 3 × 4 h/week using polysulfone membrane (Fresenius Medical Care AG, Bad Homburg, Germany). For the heparin-based iHD, patients received unfractionated heparin at an initial dose of 30 U/kg followed by continuous infusion at 7 U/kg/h. For the RCA protocol, sodium citrate 4% (Fresenius Medical Care AG, Bad Homburg, Germany) was applied immediately downstream from the connection between the dialysis catheter and arterial line of the circuit at an infusion rate calculated according to the systemic ionized calcium levels and blood flow velocity at the beginning of dialysis similar to the protocol described by Leroy et al. [12]. The target ionized calcium concentration was set at 0.7 mmol/L. Calcium-containing dialysate (1.0 mmol/L) was used for calcium supplementation, and ionized calcium values were monitored at least one hour after the start and at the end of a dialysis session.

### 4.3. Sample and Data Collection

EDTA whole blood (approx. 40 mL) samples were drawn before (before anticoagulation) and after the dialysis session. An aliquot of 5 mL whole blood was reserved for flow cytometric analysis. The remaining blood was centrifuged at 400× *g* for 10 min at 22 °C, and plasma was immediately collected, apportioned into 0.5 mL aliquots, and frozen at −80 °C until further analysis. Blood cells were further processed to monocyte isolation as described below. Routine laboratory exams and biochemical tests were performed in the Centre for Laboratory Medicine, University Hospital Muenster. Detailed demographic, laboratory, and clinical data were obtained from a computerized database that includes all dialysis patients’ clinical and biological characteristics. We used the Chronic Kidney Disease—Epidemiology (CKD-EPI) equation to estimate the glomerular filtration rate (eGFR).

### 4.4. Characterization of Monocytes in Whole Blood Using Flow Cytometry

Two aliquots of 100 μL EDTA whole blood were incubated in the presence or not of 2 ng/mL lipopolysaccharide (LPS) for 4 h at 37 °C and, subsequently, stained with a panel of antibodies for the identification of monocyte activation markers (Table S3). Fluorochrome-labeled anti-human antibodies used for flow cytometry were purchased from Biolegend (San Diego, CA, USA) and are specified in Table S3. Briefly, whole blood was incubated with premixed antibodies (final dilution of each antibody at 1:200) for 30 min in the dark at 4 °C and treated with 2 mL of lyse/fix buffer (ddH_2_O containing 1% paraformaldehyde and 3% di-ethylene glycol) for 15 min at room temperature to lyse red blood cells. The remaining cells (leukocytes) were pelleted at 500 g for 5 min at 22 °C and resuspended in 500 μL PBS containing 1% paraformaldehyde afterward. A NAVIOS EX (Beckman Coulter, Indianapolis, IN, USA) 10-color flow cytometer was used to acquire 200.000 events, and FlowJo Data Analysis Software (https://www.flowjo.com/solutions/flowjo, accessed on 26 January 2024, BD Biosciences, San Jose, CA, USA) was used for analysis. The compensation between colors was performed using single-stained beads (OneCompe eBeads, eBiosciences, San Diego, CA, USA) and FlowJo. The total leukocyte population was identified by plotting CD45 against SSC-A within the singlets. Monocytes (CD45^+^CD14^+^ cells) were further characterized according to the expression levels of HLA-DR, CD80, and CD86 given as mean fluorescence intensity (MFI).

### 4.5. Monocyte Isolation

After plasma separation, blood cells were diluted 1:2 (*v*:*v*) in PBS, and peripheral blood mononuclear cells were enriched by density gradient centrifugation on Pancoll human (Density 1.077 g/mL; PAN Biotech, Aidenbach, Germany). Monocytes were further isolated using the Pan Monocyte Isolation Kit II according to the manufacturer’s instructions (Miltenyi Biotec, Bergisch Gladbach, Germany). Unlabelled monocytes were resuspended in RPMI 1640 containing 15% fetal calf serum (FCS), 1% nonessential amino acids, 1% Penicillin/Streptomycin, and 1% L-Glutamine and seeded at a density of 1 × 10^6^ cell/mL in Teflon culture bags (Iumox film; Sarstedt, Nümbrecht, Germany). After overnight rest at 37 °C and 7% CO_2_, bags were placed on ice for 30 min, and cells were (detached by gently tapping the cooled bags and) transferred to a 50 mL Falcon tube and centrifuged at 300× *g* for 7 min at 4 °C. Cell pellets were lysed with 350 µL RNA lysis buffer containing β-mercaptoethanol and frozen at −80 °C until RNA isolation and gene expression analysis as described below.

### 4.6. Determination of Cytokines and Circulating Proteins

Plasma was diluted 1:2, and inflammatory cytokines [Angiopoietin-2, interleukins (IL) 1β, 4 and 6, interferon γ (IFNγ), Fractalkine/CX3CL1, and monocyte chemoattractant protein-1 (MCP-1) were measured using the LEGENDplex bead array (human custom panel) following the manufacturer’s instructions (Biolegend, San Diego, CA, USA). Flow cytometry data were acquired using a FACSCalibur (Becton-Dickinson, Heidelberg, Germany) and analyzed using the LEGENDplex™ Data Analysis Software Suite (https://www.bioz.com/result/legendplex%20software/product/BioLegend, accessed on 26 January 2024, Biolegend, San Diego, CA, USA). Commercial ELISA kits were used to determine the plasma levels of fibroblast growth factor (FGF)-23 (DY2604; R&D Systems, Minneapolis, MN, USA), IL-10 (900-M21; Peprotech, Cranbury, NJ, USA), β-defensins (BD-1/900-M202, BD-2/900-M172, BD-3/900-M210; Peprotech, Cranbury, NJ, USA), and cathelicidin (LL-37/HK321-02; Hycultec, Beutelsbach, Germany). All samples were measured in duplicate according to the manufacturer’s instructions.

### 4.7. RNA Isolation and Real-Time Quantitative PCR

Total RNA was isolated from monocytes using the RNEasy Mini Kit (Qiagen, Hilden, Germany). The RNA integrity was assessed with an Agilent 2100 Bioanalyzer (Agilent Technologies, Santa Clara, CA, USA). Quality measurements were performed using RNA ScreenTape and Reagents on TapeStation (all from Agilent Technologies, Santa Clara, CA, USA). Only high-quality (RIN > 9.0) total RNA samples were further processed. RNA quality control and real-time analysis were performed at the Core Facility Genomics, University Hospital Münster, Germany.

For real-time PCR analysis, two micrograms of RNA were reverse transcribed with the M-MLV reverse transcriptase (Agilent Technologies, Santa Clara, CA, USA) in a 20 µL reaction volume. cDNA was diluted at 1:20, and real-time PCR was carried out using the SYBR Select Master Mix for CFX (Applied Biosystems, Waltham, MA, USA) with the ABI PRISM 7700 Sequence Detection System (Applied Biosystems, Waltham, MA, USA). The optimum annealing temperature was 60 °C, and the reaction procedure was as follows: pre-denaturation at 95 °C for 2 min followed by 40 cycles of 95 °C for 15 s and 60 °C for 1 min. Melting curve analysis was performed from 60 °C to 95 °C, 0.5 °C per 5 s increments. The relative gene expression values were evaluated using the 2^−ΔΔCt^ method and 18S as a reference gene. Primer sequences are HLA-DR forward 5′-ctcttctcaagcactgggagttt-3′ and reverse 5′-atgaagatggtcccaataatgatg-3′; MCP-1 forward 5′-tgcagaggctcgcgagcta-3′ and reverse 5′-caggtggtccatggaatcctga-3′; TLR4 forward 5′-cgaggaagagaagacaccagt-3′ and reverse 5′-catcatcctcactgcttctgt-3′; and 18S forward 5′-ctcaacacgggaaacctcac-3′ and reverse 5′-cgctccaccaactaagaacg-3′. Results were log-transformed before statistical analysis.

### 4.8. RNA Sequencing

RNA sequencing (RNA-seq) libraries were prepared from 570 ng total RNA using the NEBNext Ultra II RNA directional Kit, and single-read sequencing was performed using a NextSeq 2000 System (Illumina, San Diego, CA, USA) with a read length of 72 bp. Using a molecular barcode, the samples were demultiplexed (bcl convert 3.8.4) to fastQ data and quality controlled (FastQC v. 0.11.9). Trimmomatics were used for adapter trimming and read filtering. The resulting reads were aligned to the reference genome using Hisat22 (v. 2.1.0). The aligned reads were sorted using SAMtools (v. 1.9). The sorted and aligned reads were counted into genes using HTSeq counts. Differential gene expression analysis, principal component analysis (PCA), and volcano plots were performed using the r-Package DESeq2 (v. 1.32.0). Sequencing and bioinformatics were performed at the Core Facility Genomics, University Hospital Münster, Germany.

### 4.9. Functional Enrichment Analysis

Functional enrichment analysis for the differentially expressed genes (log2-fold expression >1.5 and FDR adjusted *p*-value < 0.05) was performed with ENRICHR at https://maayanlab.cloud/Enrichr/ (accessed on 6 September 2023) [44,45]. We selected the GO Biological Process 2023 and the signaling pathways Reactome 2022 and KEGG 2021 Human. The top enriched terms were plotted as bar charts sorted by the adjusted *p*-valued ranking (plotted as −log_10_ adjusted *p*-value). REVIGO (REduce + VIsualize Gene Ontology) web tool version 1.8.1 (http://revigo.irb.hr/; accessed on 6 September 2023) was used for clustering GO terms based on their semantic similarity (SimRel), *p*-values, and relatedness. Only GO terms with adjusted *p*-value < 0.05 by ENRICHR analysis were used. We selected Homo sapiens (9606) in advanced options and removed obsolete GO terms (default) [46]. Complementary analyses were performed using Ingenuity Pathway Analysis in Ingenuity Pathway Analysis (IPA; Qiagen, Hilden, Germany) [47].

### 4.10. THP-1 Cells

THP-1 monocytes (monocyte cell line; ATCC TIB-202) were grown in culture medium (RPMI 1640—phosphate content at 5.6 mM [48]—containing 10% FCS, 1% Penicillin/Streptomycin, 1% L-Glutamine, and 50 μM β-Mercaptoethanol) at 37 °C and 5% CO_2_. Three days before gene expression analysis, cells were counted, centrifuged at 200× *g* for 5 min, and resuspended at a density of 0.2 × 10^6^ cell/mL in RPMI 1640 without L-Glutamine and phosphate (MP Biomedicals, Santa Ana, CA, USA) supplemented with 10% FCS, 1% Penicillin/Streptomycin, 1% L-Glutamine, 50 μM β-Mercaptoenthanol, and 1 mM or 5 mM phosphate by adding sodium phosphate (1.0 M Na^+^; 0.6 M PO_4_^2−^). After three days of culture (24-well plate) in the same medium, cells were transferred to 1.5 mL tubes and centrifuged. Cell pellets were lysed with 350 µL RNA lysis buffer containing β-mercaptoethanol and frozen at −80 °C until RNA isolation and gene expression analysis as described above.

### 4.11. Statistical Analysis

Data were analyzed using SPSS Statistics version 27 (Table 1, patients’ characteristics) or GraphPad Prism version 9.5.1, both for Windows. Samples were assessed for normality using the Kolmogorov–Smirnov test or skewness and kurtosis (small sample size). The statistical tests used are given in individual Figure and Table legends. All analyses were considered exploratory. Accordingly, *p*-values are given as descriptive measures, and the two-sided *p* < 0.05 is considered statistically significant.

## 5. Conclusions

Our results show the differential modulation of monocytes upon exposure to RCA and SHA in end-stage renal disease patients undergoing intermittent hemodialysis. The decreased expression of activation surface markers, particularly HLA-DR, on the monocytes of patients receiving RCA indicates a temporary state of immune incompetence during and immediately after a dialysis session. Furthermore, plasma changes and transcriptomics of pre-dialysis monocytes signalize the lasting nature of the RCA-related effects, suggesting that monocytes are affected even beyond the dialysis session. In summary, our findings demonstrate that RCA—but not SHA—impairs the response of monocytes to activation stimuli and alters the immune status of these patients with potential clinical implications.

## Figures and Tables

**Table 1:** Demographics and clinical characteristics of ESRD patients receiving iHD with regional citrate anticoagulation (RCA) or systemic heparin anticoagulation (SHA).

	RCA	SHA	*p*-Value
	n = 6–12 *	n = 22–26 **	
Demographics			
Age, in years	60 ± 17	62 ± 15	0.82
Sex, male	8 (67)	18 (69)	0.87
BMI	28 ± 6	30 ± 5	0.10
Blood analysis			
Albumin, g/dL	3.1 (3.0–3.5)	3.2 (3.0–3.7)	0.57
BUN, mg/dL	38 ± 16	41 ± 15	0.47
Creatinine, mg/dL	6.2 ± 3.1	6.3 ± 2.8	0.93
CRP, mg/dL	1.4 ± 0.9	2.4 ± 1.9	0.11
Calcium, mmol/L	2.2 (2.1–2.2)	2.3 (2.1–2.4)	0.08
Ionized calcium, mmol/L	1.13 ± 0.08	1.15 ± 0.08	0.392
Phosphate, mg/dL	4.3 ± 1.3	5.8 ± 1.8	0.02
PTH, pmol/L	91 (55–106)	120 (52–242)	0.43
1,25(OH)_2_D, pg/mL	20 (10–28)	15 (10–26)	0.37
Comorbidities and risk factors			
CVD	12 (46)	8 (67)	0.24
Diabetes	12 (46)	4 (33)	0.46
Hypertension	22 (85)	12 (100)	0.15
Smoking	8 (31)	3 (25)	0.72
Dialysis			
Weekly Kt/V	1.4 ± 0.3	1.4 ± 0.3	0.64
Session duration, hours	4	4	-
Membrane	polysulfone high flux	polysulfone high flux	-

Continuous data are expressed as mean ± SD (normally distributed) or median (interquartile range) (non-normally distributed) and analyzed by the independent samples *t*-test or Mann–Whitney U test, respectively. Categorical variables are presented as absolute numbers (n) and percentages (%) and compared between independent samples using chi-square tests. * Six patients with missing albumin levels and one with missing CRP levels in the citrate group. ** Four patients with missing albumin levels in the heparin group. 1,25(OH)_2_D, 1,25-Dihydroxyvitamin D; BMI, body mass index; BUN, blood urea nitrogen; CRP, C-reactive protein; CVD, cardiovascular disease; ESRD, end-stage renal disease; iHD, intermittent hemodialysis; PTH, parathormone.

**Table 2:** Cytokines, antimicrobial peptides, and endothelial-related biomarkers in plasma from ESRD patients receiving iHD with regional citrate anticoagulation (RCA) or systemic heparin anticoagulation (SHA).

	RCA	SHA	Control	*p*-Value
	n = 9–12 *	n = 19–26 **	n = 22–25 ***	
Cytokines				
CX3CL1	610 (412–1086)	663 (484–842)	663 (479–811)	0.96
IFNγ	9 (5–25)	7 (2–24)	12 (7–27)	0.47
IL-1β	7 (3–16)	3 (1–9)	7 (2–10)	0.22
IL-4	3 (2–9)	2 (2–6)	4 (5–7)	0.48
IL-6	11 (8–21) ^#^	21 (7–40) ^#^	4 (3–8)	<0.001
IL-10	313 (217–431)	364 (319–571)	396 (308–588)	0.19
MCP-1	260 (163–361) ^&,§^	747 (557– 1077) ^#^	512 (353–590)	<0.0001
Antimicrobial peptides				
BD1	1077 ± 134 ^#^	976 ± 163 ^#^	1441 ± 62	<0.001
BD2	299 ± 70	381 ± 137	303 ± 217	0.54
BD3	48 (29–114) ^$^	88 (65–142) ^#^	48 (35–86)	0.004
LL-37	4.5 (3.7–4.9)	4.7 (4.0–6.0)	4.7 (4.5–7.0)	0.22
CVD and endothelium-related biomarkers				
Angio-2	3605 (2168–6451) ^#^	4468 (2117–8116) ^#^	928 (649–1464)	<0.001
FGF-23	3287 ± 2248 ^#,§^	6418 ± 3573 ^#^	1207 ± 1031	<0.001

Blood samples were collected at pre-dialysis just before anticoagulation. All results are given in pg/mL and expressed as mean ± SD (normally distributed) or median (interquartile range) (non-normally distributed). One-way analysis of variance or a Kruskal–Wallis with Dunn’s multiple comparison test was applied, respectively. ^#^ *p* < 0.05 or ^&^ *p* = 0.055 vs. control; ^§^ *p* < 0.05 or ^$^ *p* = 0.06 vs. SHA. * Three patients with missing CX3CL1, IL-1β, and IL-4 levels in the citrate group; ** three patients missing CX3CL1 levels and six patients missing and IL-1β and IL-4 levels in the heparin group. *** One patient missing IL-1β levels and three missing IL-4 levels in the control group. Angio-2, angiopoietin-2; BD, β-defensin; CVD, cardiovascular disease; CX3CL1, C-X3-C motif chemokine ligand 1/fraktalkine; ESRD, end-stage renal disease; iHD, intermittent hemodialysis; IL, interleukin; LL-37, cathelicidin; MCP-1, monocyte chemoattractant protein-1.

## Data Availability

All data generated or analyzed during this study are included in the manuscript and supporting source files (Supplementary Materials Primary Data Set S1 and S2). Further inquiries can be directed to the corresponding author.

## Supplementary Materials

The following supporting information can be downloaded at https://www.mdpi.com/article/10.3390/ijms25052900/s1.
