# Supplementary material for: Monocytes as Targets for Immunomodulation by Regional Citrate Anticoagulation
Source: Int J Mol Sci. 2024 Mar 1;25(5):2900. doi: 10.3390/ijms25052900 (PMC10932113; doi:10.3390/ijms25052900)
Supplement: Supplementary file 1 [file ijms-25-02900-s001.zip › Supplementary Material_Di Marco et al.docx]

Supplementary Material

# Monocytes as targets for immunomodulation by regional citrate anticoagulation

**Giovana S. Di Marco^1,*^, Achmet I. Chasan^2^, Göran R. Boeckel^1^, Katrin Beul^1^, Hermann Pavenstädt^1^, Johannes Roth^2^, Marcus Brand^1^**

^1^Department of Internal Medicine D, University Hospital Muenster, 48149 Muenster, Germany

^2^Institute of Immunology, University of Muenster, Muenster, 48149 Muenster, Germany

***** Correspondence: dimarco@uni-muenster.de; Tel.: +49 251 8356911

**Supplementary Materials:**

**Table S1**: Top enriched Gene Ontology hits for Biological Process 2023 in pre-dialysis monocytes isolated from ESRD patients receiving iHD with regional citrate anticoagulation.

**Table S2**: Top enriched Gene Ontology hits for Biological Process 2023 in pre-dialysis monocytes isolated from ESRD patients receiving iHD with systemic heparin anticoagulation.

**Table S3:** List of antibodies used for flow cytometry.

**Figure S1**: Enriched Reactome 2022 pathways for the differentially expressed genes (log2-fold expression > 1.5 and adjusted p-value < 0.05) in patient monocytes relative to control. The terms are displayed based on the –log10(adjusted p-value) according to the data obtained from ENRICHR analysis. The dashed line indicates the statistical significance threshold (adjusted *P*-value < 0.05). RCA, regional citrate anticoagulation; SHA, systemic heparin anticoagulation.

**Table S1:** Top enriched Gene Ontology hits for Biological Process 2023 in pre-dialysis monocytes isolated from ESRD patients receiving iHD with regional citrate anticoagulation.

| **GO Term ID** | **Name** | **-log_10_**  **(*Q*-value)** | **Genes** |
| --- | --- | --- | --- |
| **GO:0042742** | defense response to bacterium | 10.55 | ANXA3;DEFA4;STAB2;DEFA3;RNASE3;FPR2;AZU1;RNASE2;MPO;IL6;SLPI;IL1B;LCN2;S100A12;CTSG;BPI;  PGLYRP1;S100A9;ELANE;CAMP;S100A8;LTF |
| *GO:0071222* | *cellular response to lipopolysaccharide* | 4.26 | IL6;CXCL8;DEFA4;IL1B;CCL3;DEFA3;CTSG;BPI;PPBP;TNF;PF4 |
| *GO:0032496* | *response to lipopolysaccharide* | 3.35 | IL6;CXCL8;SLPI;DEFA4;IL1B;DEFA3;CTSG;PPBP;S100A9;S100A8;PF4 |
| *GO:0002237* | *response to molecule of bacterial origin* | 2.83 | IL6;CXCL8;SLPI;IL1B;CD24;S100A9;S100A8 |
| *GO:0031663* | *lipopolysaccharide-mediated signaling pathway* | 2.43 | IL1B;CCL3;BPI;TNF |
| *GO:0071219* | *cellular response to molecule of bacterial origin* | 2.39 | IL6;CXCL8;DEFA4;IL1B;DEFA3;CTSG;PPBP;PF4 |
| **GO:0019730** | antimicrobial humoral response | 8.73 | CXCL8;DEFA4;DEFA3;RNASE3;AZU1;PPBP;SLPI;S100A12;PRTN3;CTSG;PGLYRP1;S100A9;CAMP;LTF;PF4 |
| *GO:0061844* | *antimicrobial humoral immune response mediated by antimicrobial peptide* | 6.65 | CXCL8;DEFA4;S100A12;DEFA3;RNASE3;PPBP;PGLYRP1;S100A9;CAMP;LTF;PF4 |
| *GO:0019731* | *antibacterial humoral response* | 4.55 | SLPI;DEFA4;DEFA3;CTSG;RNASE3;RNASE2;CAMP;LTF |
| **GO:0006954** | inflammatory response | 6.98 | ORM1;CXCL8;CCL3L1;TNFAIP6;FPR1;HP;ADM;FPR2;AZU1;PPBP;TNF;TPST1;IL6;IL1B;CCL3;CHI3L1;S100A9;S100A8;PF4 |
| **GO:0050832** | defense response to fungus | 6.16 | CLEC4D;DEFA4;CLEC6A;S100A12;CLEC4E;S100A9;S100A8;LTF |
| *GO:0061760* | *antifungal innate immune response* | 1.78 | CLEC4D;CLEC6A;CLEC4E |
| **GO:0050829** | defense response to Gram-negative bacterium | 6.13 | IL6;DEFA4;DEFA3;CTSG;BPI;RNASE3;AZU1;RNASE2;CAMP;ELANE;LTF |
| *GO:0050830* | *defense response to Gram-positive bacterium* | 4.64 | IL6;IL1B;DEFA4;STAB2;DEFA3;CTSG;RNASE3;PGLYRP1;RNASE2;CAMP |
| **GO:0044839** | cell cycle G2/M phase transition | 5.11 | CCNA2;MELK;CDK1;FOXM1;PKMYT1;CDC25C;AURKB;CDC25A |
| *GO:0000086* | *G2/M transition of mitotic cell cycle* | 4.28 | CCNA2;MELK;CDK1;FOXM1;PKMYT1;CDC25C;CDC25A |
| *GO:0044772* | *mitotic cell cycle phase transition* | 3.11 | CCNA2;MELK;CCNE2;CDK1;CDC25C;PKMYT1;FOXM1;CDC25A;CDKN3 |
| *GO:0044843* | *cell cycle G1/S phase transition* | 1.38 | CCNA2;CCNE2;CDC25A;CDKN3 |
| **GO:1990266** | neutrophil migration | 5.01 | CXCL8;KLF5;CCL3L1;CCL3;S100A12;PRTN3;PPBP;S100A9;S100A8;PF4 |
| *GO:0030593* | *neutrophil chemotaxis* | 4.47 | CXCL8;KLF5;CCL3L1;CCL3;S100A12;PPBP;S100A9;S100A8;PF4 |
| *GO:0071621* | *granulocyte chemotaxis* | 4.34 | CXCL8;KLF5;CCL3L1;CCL3;S100A12;PPBP;S100A9;S100A8;PF4 |
| *GO:0002548* | *monocyte chemotaxis* | 3.07 | IL6;FLT1;CCL3L1;CCL3;S100A12;CTSG |
| **GO:0002227** | innate immune response in mucosa | 4.55 | DEFA4;DEFA3;RNASE3;RNASE2;CAMP;LTF |
| *GO:0002385* | *mucosal immune response* | 3.96 | DEFA4;DEFA3;RNASE3;RNASE2;CAMP;LTF |
| **GO:0007088** | regulation of mitotic nuclear division | 4.26 | CDCA2;EGF;IL1B;CHEK1;NEK2;MKI67;CDC25C;PKMYT1 |
| *GO:0007094* | *mitotic spindle assembly checkpoint signaling* | 2.15 | NUF2;BUB1B;TTK;SPC24 |
| *GO:0071173* | *spindle assembly checkpoint signaling* | 2.15 | NUF2;BUB1B;TTK;SPC24 |
| *GO:0071174* | *mitotic spindle checkpoint signaling* | 2.15 | NUF2;BUB1B;TTK;SPC24 |
| *GO:0045841* | *negative regulation of mitotic metaphase/anaphase transition* | 2.06 | NUF2;BUB1B;TTK;SPC24 |
| *GO:1901970* | *positive regulation of mitotic sister chromatid separation* | 1.84 | BIRC5;AURKB;DLGAP5 |
| *GO:0033047* | *regulation of mitotic sister chromatid segregation* | 1.38 | CDK1;AURKB |
| **GO:1902850** | microtubule cytoskeleton organization involved in mitosis | 4.12 | KIF4A;NUF2;CDK1;BIRC5;TTK;KIF11;DLGAP5;AURKB |
| *GO:0000070* | *mitotic sister chromatid segregation* | 3.11 | SGO1;TPX2;KIF18B;SPAG5;KIFC1;CDCA5;NCAPG;KIF11;DLGAP5 |
| *GO:0090307* | *mitotic spindle assembly* | 2.98 | TPX2;KIFC1;KIF4A;BIRC5;KIF11;AURKB |
| *GO:0000819* | *sister chromatid segregation* | 2.56 | TOP2A;SGO1;KIF18B;SPAG5;KIFC1 |
| *GO:0140014* | *mitotic nuclear division* | 1.52 | SGO1;KIF18B;SPAG5;KIFC1 |
| *GO:0007080* | *mitotic metaphase plate congression* | 1.41 | CDT1;KIFC1;CDCA5;NUF2 |
| **GO:0007052** | mitotic spindle organization | 3.93 | TPX2;KIFC1;KIF4A;NUF2;BIRC5;TTK;KIF11;DLGAP5;AURKB |
| **GO:0050727** | regulation of inflammatory response | 3.68 | TNFAIP6;ACOD1;FPR2;MMP8;TNF;IL6;IL1B;CCL3;S100A12;GHRL;S100A9;ELANE;S100A8;PTGES |
| *GO:0050729* | *positive regulation of inflammatory response* | 2.60 | IL6;IL1B;CCL3;S100A12;MMP8;TNF;S100A9;S100A8 |
| *GO:0031349* | *positive regulation of defense response* | 2.29 | IL1B;CCL3;ACOD1;S100A12;FPR2;TNF;S100A9;S100A8 |
| *GO:0032103* | *positive regulation of response to external stimulus* | 1.84 | IL1B;CCL3;ACOD1;S100A12;FPR2;TNF;S100A9;S100A8 |
| **GO:0051783** | regulation of nuclear division | 3.59 | CDCA2;NEK2;CDC25C;MKI67;PKMYT1 |
| **GO:0032680** | regulation of tumor necrosis factor production | 3.41 | IL6;ORM1;CCL3;BPI;GHRL;AZU1;MMP8;CLU;LTF;PF4 |
| *GO:0032760* | *positive regulation of tumor necrosis factor production* | 2.58 | ORM1;IL6;CCL3;AZU1;MMP8;CLU;PF4 |
| *GO:1903557* | *positive regulation of tumor necrosis factor superfamily cytokine production* | 2.49 | ORM1;IL6;CCL3;AZU1;MMP8;CLU;PF4 |
| *GO:1903556* | *negative regulation of tumor necrosis factor superfamily cytokine production* | 1.43 | ORM1;BPI;GHRL;LTF |
| **GO:0150078** | positive regulation of neuroinflammatory response | 3.11 | IL6;IL1B;MMP8;TNF |
| *GO:0002675* | *positive regulation of acute inflammatory response* | 1.89 | IL6;IL1B;TNF |
| **GO:0045765** | regulation of angiogenesis | 3.10 | CEACAM1;IL6;SPARC;LRG1;SEMA6A;CXCL8;FLT1;ANXA3;IL1B;CHI3L1;GHRL;PF4 |
| *GO:1904018* | *positive regulation of vasculature development* | 1.56 | CXCL8;FLT1;LRG1;ANXA3;IL1B;CHI3L1 |
| *GO:0045766* | *positive regulation of angiogenesis* | 1.38 | CXCL8;FLT1;LRG1;ANXA3;IL1B;CHI3L1 |
| *GO:0016525* | *negative regulation of angiogenesis* | 1.35 | SPARC;SEMA6A;GHRL;TNF;PF4 |
| **GO:0001818** | negative regulation of cytokine production | 2.99 | IL6;ORM1;CEACAM1;CR1;ACOD1;BPI;GHRL;INHBA;MMP8;TNF;ELANE |
| **GO:0010564** | regulation of cell cycle process | 2.66 | CDCA2;SPAG5;CHEK1;NEK2;KIF11;MKI67;CDC25C;PKMYT1;AURKB |
| *GO:0007095* | *mitotic G2 DNA damage checkpoint signaling* | 2.73 | DEPDC1B;CHEK1;CDK1;CLSPN;TICRR |
| *GO:0000076* | *DNA replication checkpoint signaling* | 2.60 | CDT1;CDC45;CLSPN;TICRR |
| *GO:0010948* | *negative regulation of cell cycle process* | 2.56 | CHEK1;NEK2;AURKB;E2F7;E2F8 |
| *GO:0090068* | *positive regulation of cell cycle process* | 2.39 | SPAG5;EGF;IL1B;NCAPG;BIRC5;AURKB;E2F7;E2F8 |
| *GO:1901992* | *positive regulation of mitotic cell cycle phase transition* | 2.36 | RRM2;CDCA5;CDK1;CDC25C;DLGAP5;CDC25A |
| *GO:0031570* | *DNA integrity checkpoint signaling* | 2.29 | CDT1;CDC45;CHEK1;RPA4;CLSPN |
| *GO:0044818* | *mitotic G2/M transition checkpoint* | 2.15 | DEPDC1B;CHEK1;CDK1;CLSPN;TICRR |
| *GO:0044773* | *mitotic DNA damage checkpoint signaling* | 1.88 | DEPDC1B;CHEK1;CDK1;CLSPN;TICRR |
| *GO:0010971* | *positive regulation of G2/M transition of mitotic cell cycle* | 1.54 | CDK1;CDC25C;CDC25A |
| *GO:1902751* | *positive regulation of cell cycle G2/M phase transition* | 1.44 | CDK1;CDC25C;CDC25A |
| *GO:0007093* | *mitotic cell cycle checkpoint signaling* | 1.40 | DEPDC1B;CHEK1;AURKB |
| *GO:0007346* | *regulation of mitotic cell cycle* | 1.32 | CDCA2;NEK2;MKI67;CDC25C;PKMYT1;DLGAP5 |
| *GO:0010972* | *negative regulation of G2/M transition of mitotic cell cycle* | 1.32 | DEPDC1B;CHEK1;AURKB |
| *GO:0033314* | *mitotic DNA replication checkpoint signaling* | 1.32 | CLSPN;TICRR |
| **GO:0043410** | positive regulation of MAPK cascade | 2.65 | FLT1;CCL3L1;EGF;FPR2;MMP8;TNF;IL6;IL1B;CCL3;S100A12;CHI3L1;GHRL;CD24;ELANE |
| *GO:0070372* | *regulation of ERK1 and ERK2 cascade* | 1.38 | CCNA2;CEACAM1;SEMA6A;CCL3L1;IL1B;CCL3;CHI3L1;FPR2;TNF |
| **GO:1904029** | regulation of cyclin-dependent protein kinase activity | 2.60 | CCNA2;CCNE2;CDC25C;PKMYT1;CDC25A;CDKN3 |
| *GO:0071900* | *regulation of protein serine/threonine kinase activity* | 2.46 | IL6;IL1B;MMP8;TNF |
| *GO:0001934* | *positive regulation of protein phosphorylation* | 2.43 | EGF;ITGB3;LIMK2;TTK;FPR2;AZU1;INHBA;TNF;AURKB;CCNA2;IL6;IL1B;CLEC6A;BIRC5;CHI3L1 |
| *GO:0043405* | *regulation of MAP kinase activity* | 2.43 | FLT1;EGF;IL1B;S100A12;TRIB3;CD24;TNF;ELANE |
| *GO:0071902* | *positive regulation of protein serine/threonine kinase activity* | 2.39 | FLT1;EGF;IL1B;S100A12;CD24;TNF;ELANE;LTF |
| *GO:0043406* | *positive regulation of MAP kinase activity* | 2.33 | FLT1;EGF;IL1B;S100A12;CD24;TNF;ELANE |
| *GO:0000079* | *regulation of cyclin-dependent protein serine/threonine kinase activity* | 1.98 | CCNA2;CCNE2;CDC25C;PKMYT1;CDC25A;CDKN3 |
| *GO:0045860* | *positive regulation of protein kinase activity* | 1.96 | CCNA2;TPX2;ITGB3;CLSPN;AZU1;FCGR1A;CLU;LTF |
| *GO:0042327* | *positive regulation of phosphorylation* | 1.86 | CCNA2;FLT1;EGF;IL1B;ITGB3;LIMK2;BIRC5;FPR2;TNF;AURKB |
| *GO:0031401* | *positive regulation of protein modification process* | 1.54 | CCNA2;CDCA2;IL1B;ITGB3;LIMK2;BIRC5;FPR2;TNF;AURKB |
| **GO:0050995** | negative regulation of lipid catabolic process | 2.56 | HCAR2;IL1B;TNF;PLIN5 |
| **GO:0070486** | leukocyte aggregation | 2.56 | IL1B;S100A9;S100A8 |
| **GO:0150077** | regulation of neuroinflammatory response | 2.49 | IL6;IL1B;MMP8;TNF |
| **GO:0060251** | regulation of glial cell proliferation | 2.43 | IL6;IL1B;TNF |
| **GO:0046602** | regulation of mitotic centrosome separation | 2.43 | CHEK1;NEK2;KIF11 |
| **GO:0010883** | regulation of lipid storage | 2.39 | IL6;ITGB3;TNF;PLIN5 |
| **GO:0051240** | positive regulation of multicellular organismal process | 2.39 | ACSL1;EGF;G0S2;INHBA;TNF;HCAR2;IL6;LRG1;BAMBI;IL1B;CLEC6A;SPP1;LCN2;GHRL;B4GALT5 |
| **GO:0010721** | negative regulation of cell development | 2.34 | IL6;IL1B;RFLNB;TNF |
| **GO:1905953** | negative regulation of lipid localization | 2.33 | IL6;ITGB3;TNF |
| *GO:0010888* | *negative regulation of lipid storage* | 1.67 | IL6;ITGB3;TNF |
| **GO:0031960** | response to corticosteroid | 2.23 | IL1RN;IL6;TNF |
| *GO:0071396* | *cellular response to lipid* | 1.88 | IL6;CXCL8;IL1B;DEFA4;SPP1;ACOD1;DEFA3;CTSG;PPBP;PF4 |
| *GO:0051384* | *response to glucocorticoid* | 1.38 | IL1RN;IL6;TNF |
| **GO:0034114** | regulation of heterotypic cell-cell adhesion | 2.23 | IL1RN;CEACAM6;IL1B;TNF |
| **GO:2000105** | positive regulation of DNA-templated DNA replication | 2.23 | CDT1;E2F7;E2F8 |
| **GO:0090330** | regulation of platelet aggregation | 2.17 | CEACAM1;IL6;ALOX12;CTSG |
| *GO:0090331* | *negative regulation of platelet aggregation* | 1.38 | CEACAM1;ALOX12 |
| **GO:0043408** | regulation of MAPK cascade | 2.15 | CCNA2;CEACAM1;IL6;FLT1;EGF;IL1B;GHRL;CD24;MMP8;TNF |
| **GO:0045833** | negative regulation of lipid metabolic process | 2.15 | HCAR2;CEACAM1;IL1B;TNF |
| *GO:0050994* | *regulation of lipid catabolic process* | 1.47 | HCAR2;IL1B;TNF |
| **GO:0010631** | epithelial cell migration | 2.15 | ANLN;LRG1;S100A12;S100P;S100A9 |
| **GO:0002430** | complement receptor mediated signaling pathway | 2.15 | CR1;FPR1;FPR2 |
| *GO:0002429* | *immune response-activating cell surface receptor signaling pathway* | 1.47 | CR1;FPR1;FPR2 |
| **GO:0034501** | protein localization to kinetochore | 2.15 | CDK1;TTK;KNL1 |
| *GO:1903083* | *protein localization to condensed chromosome* | 2.23 | CDK1;TTK;KNL1 |
| *GO:0071459* | *protein localization to chromosome. centromeric region* | 1.67 | CDK1;TTK;KNL1 |
| **GO:2001239** | regulation of extrinsic apoptotic signaling pathway in absence of ligand | 2.11 | IL1B;INHBA;TNF;PF4 |
| *GO:2001240* | *negative regulation of extrinsic apoptotic signaling pathway in absence of ligand* | 1.51 | IL1B;TNF;PF4 |
| **GO:0045429** | positive regulation of nitric oxide biosynthetic process | 2.11 | IL1B;MMP8;CLU;TNF |
| *GO:1904407* | *positive regulation of nitric oxide metabolic process* | 2.06 | IL1B;MMP8;CLU;TNF |
| *GO:0045428* | *regulation of nitric oxide biosynthetic process* | 1.67 | IL1B;MMP8;CLU;TNF |
| **GO:0036230** | granulocyte activation | 2.06 | CXCL8;CTSG;CAMP |
| *GO:0042119* | *neutrophil activation* | 1.67 | CXCL8;CTSG;CAMP |
| *GO:0001774* | *microglial cell activation* | 1.59 | AZU1;CLU;TNF |
| **GO:0002762** | negative regulation of myeloid leukocyte differentiation | 1.96 | CEACAM1;TNFAIP6;CCL3;INHBA |
| *GO:0045671* | *negative regulation of osteoclast differentiation* | 1.54 | TNFAIP6;CCL3;LTF |
| *GO:0050768* | *negative regulation of neurogenesis* | 1.35 | IL6;IL1B;TNF |
| **GO:0032677** | regulation of interleukin-8 production | 1.93 | IL6;IL1B;CHI3L1;BPI;TNF;ELANE |
| **GO:0048522** | positive regulation of cellular process | 1.92 | CR1;SPAG5;EGF;HP;TTK;AZU1;FOXM1;TNF;IL6;CEACAM6;BAMBI;IL1B;CHEK1;BIRC5;PRTN3;S1PR3;S100A9;S100A8 |
| **GO:0032675** | regulation of interleukin-6 production | 1.90 | IL6;ORM1;IL1B;BPI;GHRL;MMP8;TNF |
| **GO:0034116** | positive regulation of heterotypic cell-cell adhesion | 1.89 | CEACAM6;IL1B;TNF |
| **GO:0032651** | regulation of interleukin-1 beta production | 1.88 | ORM1;IL6;CCL3;GHRL;AZU1;TNF |
| *GO:0032731* | *positive regulation of interleukin-1 beta production* | 1.86 | ORM1;IL6;CCL3;AZU1;TNF |
| *GO:0032732* | *positive regulation of interleukin-1 production* | 1.69 | ORM1;IL6;CCL3;AZU1;TNF |
| **GO:0070098** | chemokine-mediated signaling pathway | 1.86 | CXCL8;CCL3L1;CCL3;PPBP;PF4 |
| *GO:1990869* | *cellular response to chemokine* | 1.82 | CXCL8;CCL3L1;CCL3;PPBP;PF4 |
| **GO:0014070** | response to organic cyclic compound | 1.84 | IL1B;CCL3;SPP1;CLU;TNF |
| **GO:0050764** | regulation of phagocytosis | 1.84 | IL1B;PRTN3;FPR2;AZU1;TNF |
| *GO:0045807* | *positive regulation of endocytosis* | 1.44 | IL1B;FPR2;AZU1;CLU;TNF |
| **GO:0032715** | negative regulation of interleukin-6 production | 1.83 | ORM1;BPI;GHRL;TNF |
| **GO:1903140** | regulation of establishment of endothelial barrier | 1.78 | IL1B;S1PR3;TNF |
| *GO:1901550* | *regulation of endothelial cell development* | 1.38 | IL1B;TNF |
| **GO:0032757** | positive regulation of interleukin-8 production | 1.76 | IL6;IL1B;CHI3L1;TNF;ELANE |
| **GO:0006259** | DNA metabolic process | 1.72 | TOP2A;NEIL3;CDC45;EXO1;CHEK1;RPA4;PCLAF;CDK1;MCM10;TYMS;TICRR |
| **GO:1904892** | regulation of receptor signaling pathway via STAT | 1.71 | IL6;EGF;TP53INP2 |
| **GO:0051091** | positive regulation of DNA-binding transcription factor activity | 1.71 | IL6;BEX1;ANXA3;IL1B;S100A12;CLU;TNF;S100A9;S100A8;LTF |
| *GO:0051092* | *positive regulation of NF-kappaB transcription factor activity* | 1.87 | IL6;IL1B;S100A12;CLU;TNF;S100A9;S100A8;LTF |
| **GO:0008284** | positive regulation of cell population proliferation | 1.68 | CR1;EGF;TTK;FOXM1;TNF;CCNA2;IL6;CEACAM6;BAMBI;IL1B;BIRC5;PRTN3;S1PR3;ELANE;LTF |
| **GO:0000165** | MAPK cascade | 1.67 | EGF;IL1B;CDK1;CCL3;FGF13;TNF |
| **GO:0030856** | regulation of epithelial cell differentiation | 1.67 | CEACAM1;MAFF;CD24 |
| **GO:0051961** | negative regulation of nervous system development | 1.67 | IL6;IL1B;TNF |
| **GO:0071887** | leukocyte apoptotic process | 1.67 | HCAR2;IL6 |
| **GO:0070365** | hepatocyte differentiation | 1.67 | E2F7;E2F8 |
| **GO:0090594** | inflammatory response to wounding | 1.67 | IL6;TNF |
| **GO:0033690** | positive regulation of osteoblast proliferation | 1.67 | CCNA2;LTF |
| **GO:2000659** | regulation of interleukin-1-mediated signaling pathway | 1.67 | IL1RN;IL6 |
| **GO:0032466** | negative regulation of cytokinesis | 1.67 | E2F7;E2F8 |
| **GO:0043152** | induction of bacterial agglutination | 1.67 | RNASE3;RNASE2 |
| **GO:0060558** | regulation of calcidiol 1-monooxygenase activity | 1.67 | IL1B;TNF |
| **GO:0070601** | centromeric sister chromatid cohesion | 1.67 | SGO1;BUB1B |
| **GO:0140271** | hexose import across plasma membrane | 1.67 | SLC2A3;SLC2A5 |
| *GO:0008645* | *hexose transmembrane transport* | 1.51 | SLC2A3;PPBP;SLC2A5 |
| *GO:1904659* | *glucose transmembrane transport* | 1.51 | SLC2A3;PPBP;SLC2A5 |
| *GO:0015755* | *fructose transmembrane transport* | 1.44 | SLC2A3;SLC2A5 |
| **GO:0019221** | cytokine-mediated signaling pathway | 1.65 | CEACAM1;IL6;CXCL8;CCL3L1;IL1B;CCL3;CTSG;PPBP;TNF;PF4 |
| **GO:0002673** | regulation of acute inflammatory response | 1.65 | IL6;F12;PTGES |
| **GO:0006260** | DNA replication | 1.54 | CHEK1;RPA4;PCLAF;CDK1;TICRR |
| **GO:0014015** | positive regulation of gliogenesis | 1.54 | IL6;TNF |
| **GO:0006270** | DNA replication initiation | 1.54 | CDC45;RPA4;MCM10 |
| **GO:0009251** | glucan catabolic process | 1.54 | MGAM;STBD1 |
| **GO:1902412** | regulation of mitotic cytokinesis | 1.54 | BIRC5;AURKB |
| **GO:0060353** | regulation of cell adhesion molecule production | 1.54 | CXCL8;IL1B |
| **GO:0070163** | regulation of adiponectin secretion | 1.54 | HCAR2;IL1B |
| **GO:1901099** | negative regulation of signal transduction in absence of ligand | 1.51 | IL1B;TNF;PF4 |
| **GO:0042129** | regulation of T cell proliferation | 1.47 | IL6;CR1;ARG1;IL1B;CLC |
| **GO:0010718** | positive regulation of epithelial to mesenchymal transition | 1.47 | IL6;LRG1;BAMBI;IL1B |
| **GO:0035336** | long-chain fatty-acyl-CoA metabolic process | 1.47 | DGAT2;ACSL1;ELOVL7 |
| *GO:0046949* | *fatty-acyl-CoA biosynthetic process* | 1.40 | ACSL1;ELOVL7;SLC27A2 |
| **GO:0051101** | regulation of DNA binding | 1.45 | CDT1;EGF;HJURP;MMP8 |
| **GO:0007597** | blood coagulation. intrinsic pathway | 1.44 | GP9;F12 |
| **GO:0043491** | protein kinase B signaling | 1.44 | IL1B;CCL3;TNF |
| **GO:0010800** | positive regulation of peptidyl-threonine phosphorylation | 1.44 | EGF;CHI3L1;AZU1 |
| **GO:0032147** | activation of protein kinase activity | 1.41 | TPX2;TNFSF15;ITGB3;CHI3L1;CLSPN |
| **GO:0044319** | wound healing. spreading of cells | 1.40 | CCNA2;CEACAM1;LRG1 |
| **GO:2001141** | regulation of RNA biosynthetic process | 1.38 | CXCL8;IL1B |
| **GO:0002526** | acute inflammatory response | 1.38 | IL6;HP;ELANE |
| **GO:0002701** | negative regulation of production of molecular mediator of immune response | 1.38 | CR1;TNF |
| **GO:0046717** | acid secretion | 1.38 | GHRL;SLC51A |
| **GO:0051788** | response to misfolded protein | 1.38 | F12;CLU |
| **GO:0042127** | regulation of cell population proliferation | 1.38 | CXCL8;CR1;CCL3L1;EGF;TTK;INHBA;FOXM1;CLU;IL6;CEACAM6;BAMBI;IL1B;BIRC5;GHRL;PRTN3;S1PR3;E2F7;CDKN3;PF4 |
| **GO:0019722** | calcium-mediated signaling | 1.37 | CXCL8;CCL3;FPR2;AZU1;CLU |
| **GO:0070555** | response to interleukin-1 | 1.37 | CXCL8;CCL3L1;IL1B;CCL3;CHI3L1 |
| **GO:0045667** | regulation of osteoblast differentiation | 1.37 | CCNA2;IL6;TNFAIP6;BAMBI;LTF |
| **GO:0046425** | regulation of receptor signaling pathway via JAK-STAT | 1.37 | IL6;EGF;TP53INP2;TNF |
| **GO:0032722** | positive regulation of chemokine production | 1.37 | IL6;IL1B;AZU1;TNF |
| **GO:0010594** | regulation of endothelial cell migration | 1.32 | CEACAM1;SPARC;ANXA3;EGF;ITGB3 |
| **GO:0051983** | regulation of chromosome segregation | 1.32 | CDCA2;NCAPG;MKI67 |
| **GO:0045124** | regulation of bone resorption | 1.32 | IL6;ITGB3;SPP1 |
| **GO:0034654** | nucleobase-containing compound biosynthetic process | 1.32 | CDA;TYMS |
| **GO:1902680** | positive regulation of RNA biosynthetic process | 1.32 | TOP2A;IL1B |
| **GO:0018198** | peptidyl-cysteine modification | 1.32 | S100A9;S100A8 |
| **GO:0030449** | regulation of complement activation | 1.32 | CR1;IL1B |
| **GO:0051988** | regulation of attachment of spindle microtubules to kinetochore | 1.32 | SPAG5;NEK2 |
| **GO:0030949** | positive regulation of vascular endothelial growth factor receptor signaling pathway | 1.32 | IL1B;ITGB3 |
| **GO:0042770** | Signal Transduction In Response To DNA Damage | 1.32 | CHEK1;RPA4;CLSPN;E2F7 |

Q-value, adjusted *P*-value. Differentially expressed genes with a log2-fold expression >1.5 and false discovery rate (FDR)-adjusted *P*-value < 0.05 were analyzed with ENRICHR and Gene Ontology Biological Process terms with adjusted *P*-value < 0.05 were clustered by REVIGO. ESRD, end-stage renal disease; iHD, intermittent hemodialysis.

**Table S2:** Top enriched Gene Ontology hits for Biological Process 2023 in pre-dialysis monocytes isolated from ESRD patients receiving iHD with systemic heparin anticoagulation.

| **GO Term ID** | **Name** | **-log_10_**  **(*Q*-value)** | **Genes** |
| --- | --- | --- | --- |
| **GO:0042742** | defense response to bacterium | 6.10 | ANXA3;DEFA4;AZU1;MPO;SLPI;ADGRB1;LCN2;CTSG;BPI;PGLYRP1;ELANE;CAMP;LTF |
| **GO:0019730** | antimicrobial humoral response | 3.83 | SLPI;DEFA4;PRTN3;CTSG;AZU1;PGLYRP1;CAMP;LTF |
| *GO:0019731* | *antibacterial humoral response* | 2.37 | SLPI;DEFA4;CTSG;CAMP;LTF |
| **GO:0050829** | defense response to Gram-negative bacterium | 3.71 | DEFA4;CTSG;BPI;AZU1;CAMP;ELANE;LTF |
| **GO:0045765** | regulation of angiogenesis | 1.91 | SFRP1;CEACAM1;SPARC;LRG1;ANXA3;ADGRB1;CHI3L1;RAPGEF3 |
| **GO:0045840** | positive regulation of mitotic nuclear division | 1.87 | CDC20;EGF;UBE2C;NUSAP1 |
| *GO:0007088* | *regulation of mitotic nuclear division* | 1.32 | EGF;NUSAP1;MKI67;CDC25C |
| **GO:0032680** | regulation of tumor necrosis factor production | 1.57 | ORM1;BPI;AZU1;MMP8;CLU;LTF |
| **GO:0010594** | regulation of endothelial cell migration | 1.45 | CEACAM1;SPARC;ANXA3;EGF;ADGRB1 |
| **GO:0002227** | innate immune response in mucosa | 1.41 | DEFA4;CAMP;LTF |
| **GO:0010800** | positive regulation of peptidyl-threonine phosphorylation | 1.32 | EGF;CHI3L1;AZU1 |
| **GO:0000281** | mitotic cytokinesis | 1.32 | NUSAP1;KIF20A;CENPA;CEP55 |
| **GO:0050764** | regulation of phagocytosis | 1.32 | PRTN3;AZU1;OLFM4;SIRPB1 |

Q-value, adjusted *P*-value. Differentially expressed genes with a log2-fold expression >1.5 and false discovery rate (FDR)-adjusted *P*-value < 0.05 were analyzed with ENRICHR and Gene Ontology Biological Process terms with adjusted *P*-value < 0.05 were clustered by REVIGO. ESRD, end-stage renal disease; iHD, intermittent hemodialysis.

**Table S3:** List of antibodies used for flow cytometry.

| **Antibody** | **Clone** | **Host species, isotype** | **Fluorochrome** | **Dilution** |
| --- | --- | --- | --- | --- |
| CD14 | M5E2 | Mouse IgG2a, κ | Pacific Blue | 200 x |
| CD45 | HI30 | Mouse IgG1, κ | PE/Cy5 | 200 x |
| CD69 | FN50 | Mouse IgG1, κ | Alexa Fluor 700 | 200 x |
| CD80 | 2D10 | Mouse IgG1, κ | FITC | 200 x |
| CD86 | IT2.2 | Mouse IgG2b, κ | PE/Dazzle 594 | 200 x |
| HLA-DR | L243 | Mouse IgG2a, κ | APC | 200 x |

All antibodies were purchased from Biolegend (San Diego, CA, USA).

**Figure S1:** Enriched Reactome 2022 pathways for the differentially expressed genes (log2-fold expression > 1.5 and adjusted p-value < 0.05) in patient monocytes relative to control. The terms are displayed based on the –log10(adjusted p-value) according to the data obtained from ENRICHR analysis. Dashed line indicates statistical significance threshold (adjusted *P*-value < 0.05). RCA, regional citrate anticoagulation; SHA, systemic heparin anticoagulation.
